# Upper limb strength training in subacute stroke patients: study protocol of a randomised controlled trial

**DOI:** 10.1186/s13063-019-3261-3

**Published:** 2019-03-15

**Authors:** Susan Högg, Manfred Holzgraefe, Insa Wingendorf, Jan Mehrholz, Christoph Herrmann, Mark Obermann

**Affiliations:** 1 Asklepios Kliniken Schildautal, Klinik für Neurologische Rehabilitation und Klinik für Neurologische Frührehabilitation, Physiotherapie, Seesen, Germany; 2 Asklepios Kliniken Schildautal, Klinik für Neurologische Rehabilitation, Seesen, Germany; 3 Asklepios Kliniken Schildautal, Physiotherapie, Seesen, Germany; 4 Department of Public Health, Dresden Medical School, Technical University Dresden, Dresden, Germany; 5 Asklepios Kliniken Schildautal, Zentrum für Neurologie, Seesen, Germany

**Keywords:** Stroke, Rehabilitation, Upper limb, Strength, Training

## Abstract

**Background:**

Stroke patients are often affected by arm paresis, have functional impairments and receive help from professional or informal caregivers. Progressive resistance training is a common intervention for functional impairments after paresis. Randomised controlled trials (RCT) showed benefits for functional recovery after resistance training. However, there is a lack of evidence for strength training in subacute stroke patients. The aim of this study is to investigate safety and effectiveness of arm strength training in subacute stroke patients.

**Methods:**

We will conduct a prospective, assessor-blinded RCT of people with subacute stroke. We will randomly assign patients to one of two parallel groups in a 1:1 ratio and will use concealed allocation. The intervention group will receive, in addition to standard treatment, high-intensity arm training (three times per week, over three weeks; 60 min each session; with a total of nine additional sessions). The control group will receive, in addition to standard treatment, low-intensity arm training (same quantity, frequency and treatment time as the intervention group). Standard treatment for the affected arm includes mobilisation, stretching, therapeutic positioning, arm and hand motor training, strengthening exercises, mechanical assisted training, functional training and task-oriented training. The primary efficacy endpoint will be grip strength. Secondary outcome measures will be Modified Ashworth Scale, Motricity Index, Fugl-Meyer Assessment for the upper limb, Box and Block Test and Goal Attainment Scale for individual participatory goals. We will measure primary and secondary outcomes with blinded assessors at baseline and immediately after three weeks of additional therapy. Based on our sample size calculation, 78 patients will be recruited from our rehabilitation hospital in two and a half years. Drop-out rates and adverse events will be systematically recorded.

**Discussion:**

This study attempts to close the evidence gap for effects of arm strength training in subacute stroke patients. The results of this trial will provide robust evidence for effects and safety of high-intensity arm training for people with stroke.

**Trial registration:**

German Clinical Trials Register, DRKS00012484. Registered on 26 May 2017.

**Electronic supplementary material:**

The online version of this article (10.1186/s13063-019-3261-3) contains supplementary material, which is available to authorized users.

## Background

Stroke patients are often affected by decreased strength and functional impairments [1]. Paresis of the upper limb occur in 77% of the cases [1]. Muscle weakness affects activities of daily life (ADL), therefore, writing or grasping a glass of water can be difficult or even impossible [2, 3]. People with stroke often suffer from long-term impairment; consequently, after discharge from hospital, 41% of them need help for ADL and 20% receive nursing at home by family members or friends [4].

Recent studies have shown that interventions like constraint-induced movement therapy (CIMT), mirror therapy, training with virtual reality, or repetitive task training are effective to improve upper-limb function after stroke [5]. It is necessary to learn more about the effectiveness of different interventions and training methods in arm rehabilitation to increase the independence of people with stroke. The selection of training methods can be based on individual needs of patients or on their effects and the training target. Early studies showed that a greater dose of an intervention (which means the number of repetitions or the duration of intervention) seems to be an advantage for functional recovery after stroke [5]. A large amount of intervention minutes or number of repetitions are effective to increase arm function [6] and ADL [7]. There are several possibilities to vary intensity of training, e.g. through using different numbers of repetitions, various complexity of tasks, different feedback modalities, or additional weights.

Prange et al. compared robotic weight-supported arm training with a conventional training in subacute stroke patients to examine training effects on arm function and perceived motivation [8]. The robotic arm training obtained similar effects to the comparison group but with higher motivation. A higher perceived motivation can be used to increase treatment intensity or dose. In contrast to Prange et al., we will focus on effects of arm training on strength and instead of robotic weight-supported training we will use additional weight loads to increase intensity of intervention.

Chan et al. found in a single-group cohort study that weight-supported training with robotic devices can increase arm function, especially in subacute stroke patients with moderate or severe impairments [9]. The more severely affected the patients, the higher the improvements. It is unclear if robotic arm training has an advantage to another intervention type because there is no comparison group. The training effects are different in patients with mild, moderate, or severe impairments but we do not know if all these patients can train with additional weights, no matter how severe the impairments are.

Randomised controlled trials (RCT) found that resistance training for the upper limb that uses additional weights for a higher training intensity increased strength [10, 11], arm function [12–15] and arm activities [14] in chronic stroke patients. Three studies searched for adverse events (AE) after arm strength training in chronic stroke patients. Two of these found no AEs [16, 17] and one reported muscle soreness [15]. Only two studies examined treatment effects of such intensive and progressive arm training in subacute stroke patients [17, 18]. Donaldson et al. found that additional functional resistance training leads to greater improvements of arm function than standard treatment or double dose of standard treatment [18]. In this pretest–post-test clinical trial, no between-group comparisons were performed. Thus, there is no information about a possible advantage for one of the interventions in subacute stroke patients. Winstein et al. compared a control group which performed five times per week functional training with an intervention group which performed a combination of three times resistance training and two times functional training per week [19]. Mild affected stroke patients of the intervention group showed greater improvements of arm function (measured with the motor subscore of the Fugl-Meyer-Assessment for upper extremity) than the control group. There were no group differences in other functional assessments, e.g. grip strength. This study is limited by non-blinded assessors. Only these two studies investigated resistance training with additional weights in the first three month after stroke. AEs in patients in the subacute phase after stroke were not examined. This evidence related to the effectiveness of strength training (resistance training) in improving upper limb function and activities is insufficient. A solid evaluation of intensive resistance arm training in the subacute phase after stroke is not possible. It is important to investigate common interventions early after stroke to follow the recommendations of an early start to the training. An early start to the training (one week after stroke onset) with higher treatment dose speeds up the functional recovery of arm activities [20, 21]. The highest rise of recovery is achieved in the first weeks after stroke with the major part of recovery already reached at this point [20, 22]. However, less is known about which therapeutic interventions could support this early recovery. Strength training for preservation or improvement of muscle strength is recommended in stroke guidelines [23, 24]. Despite the lack of evidence for therapeutic effects and AEs in subacute and early chronic stroke patients, resistance training is recommended [25] and used in rehabilitation.

We want to compare intensive resistance arm training with low-intensity arm training in the subacute phase after stroke. We developed a training program possibly feasible for patients with mild to severe stroke. We will examine if a three-week training programme is safe and effective for these patients.

### Objective

We will use a parallel-group RCT design to assess the safety and the effects of arm strength training in persons with subacute stroke. The control group performs the same exercises with low intensity. We will evaluate arm function, activities, participation and AEs.

## Methods

### Study design

**Fig. 1:**
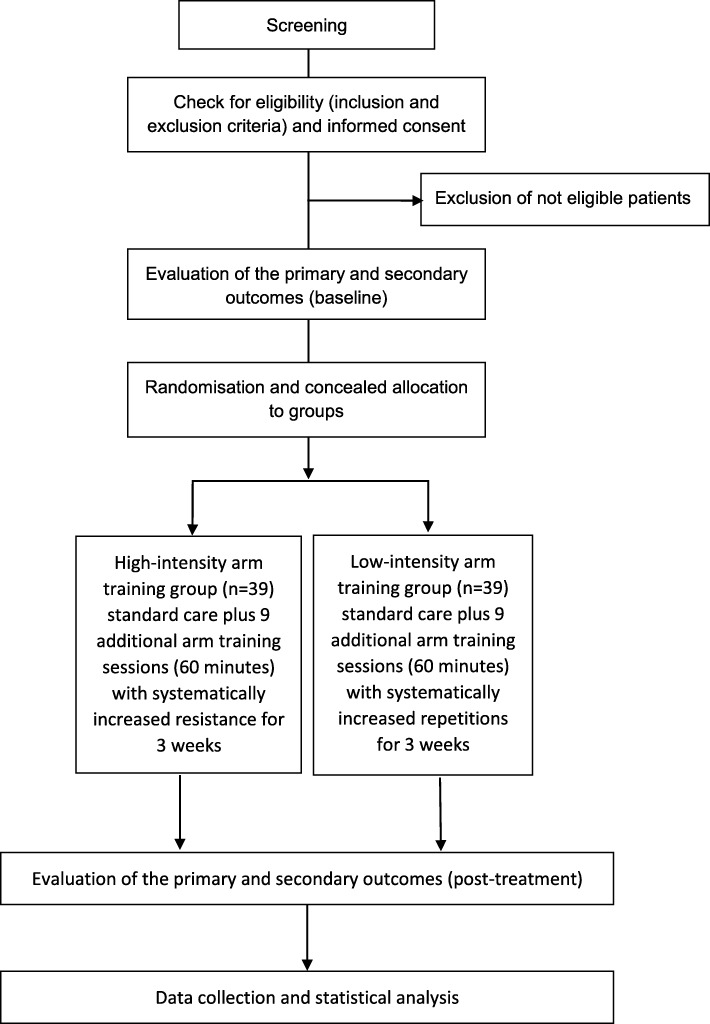
*Flow diagram* of study design

This is an assessor-blinded, parallel-group, single-centre RCT of people with upper limb paresis after stroke (see Fig. 1). Stroke was defined as ‘rapidly developing clinical signs of focal (or global) disturbance of cerebral function, with symptoms lasting 24 hours or longer […], with no apparent cause other than of vascular origin’ [26] diagnosed by a neurologist and confirmed by computer tomography or magnetic resonance imaging.

The study is conducted at the neurological rehabilitation hospital Asklepioskliniken Schildautal Seesen in Germany. Before the intervention, patients will be randomised in a 1:1 ratio into one of two intervention parallel groups.

**Fig. 2:**
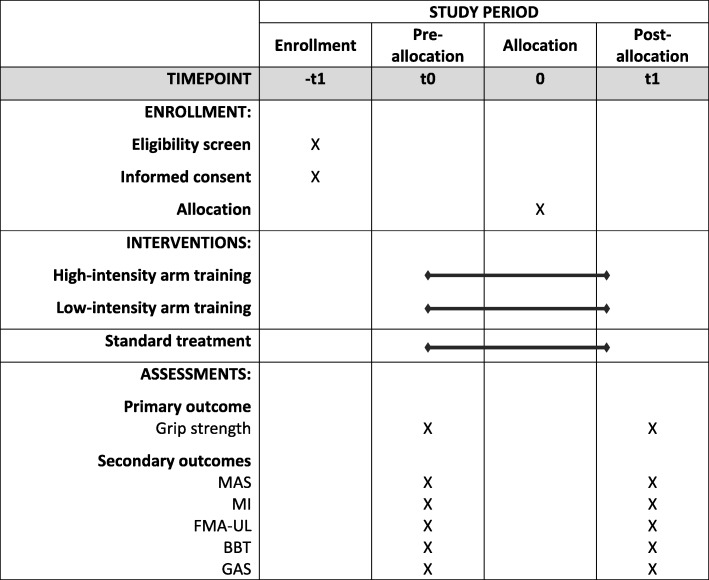
Schedule of enrolment, interventions and assessments. Time points: -t1: before baseline screening; t0: baseline; t1: 3 weeks after treatment. MAS Modified Ashworth Scale, MI Motricity Index, FMA-UL Fugl-Meyer Assessment for the upper limb, BBT Box and Block Test, GAS Goal Attainment Scale

The first experimental group will receive, in addition to standard treatment, intensive upper limb strength training with an intensity of 80% of the maximum force (three times per week, over three weeks, 60 min per session, with a total of nine additional sessions). The other group will receive, in addition to standard treatment, functional arm training with an intensity of 40% of the maximum force (with the same quantity, frequency and treatment time as the intervention group). After receiving ethical approval and registration of the study, we started recruiting in our hospital in May 2017. The final assessments will be made in 2018. The study protocol is prepared according to the SPIRIT Statement (http://www.spirit-statement.org/) [27] (see Additional file 1; for the SPIRIT figure, see Fig. 2).

### Screening process

Patient lists will be searched for potential study participants daily. We will enter all patients with the diagnosis of stroke into our screening log. Subsequently, the patient will be screened for eligibility (Fig. 2) with the inclusion and exclusion criteria as outlined in Table 1. We will fully inform eligible patients and/or their caregivers about the study; in addition, we will assign an identification number to the patient. Patients and/or their caregivers are then asked to provide informed consent. If a potential participant does not meet the inclusion criteria, we will not retain identifiable information on that patient. We will report all reasons for exclusion in a flow chart. After providing informed consent, we will record all medical and neurological information at baseline in a case report form. All baseline assessments will be conducted by an occupational therapist or a physiotherapist. Those study assessors will not be involved in treating patients or with administration of the intervention and will remain fully blinded to the patient’s group allocation throughout the whole trial.

**Table 1:** Inclusion and exclusion criteria

Inclusion criteria	
1. Participant is in the subacute phase after having a stroke (within 3 months after stroke) 2. Participant has a Barthel Index of at least 30 points 3. Participant has an arm paresis with a Medical Research Council score of 2–4 points for shoulder abduction, elbow flexion and finger flexion 4. Participant is aged > 18 years 5. Participant can lift the affected hand from their lap to a desk standing in front of them 6. Participant is able to sit for 30 s without using upper extremities for support 7. Passive range of motion is at least 90° of shoulder abduction and flexion; 90° of elbow flexion; 30° of wrist extension; 30° between pronation and supination; a touch of finger tips and thumb and submaximal finger extension is possible 8. Apraxia Screen of TULIA Score of ≥ 5 9. Written informed consent of the patient or their legal guardian has been obtained	
Exclusion criteria	
1. 4 or 5 points on Modified Ashworth Scale 2. > 5 points on a pain rating scale at rest 3. Manifest heart diseases like cardiac insufficiency (> New York Heart Association stage 1), angina pectoris, myocardial infarction within 120 days before recruitment, cardiomyopathy, hypertonia (European Society of Hypertension, grade 2 [47]) or severe cardiac arrhythmia 4. Inflammation or infection, with fibre, ECOG Performance Status Grade 3 or 4 [48] or isolation 5. Myopathy (like muscular dystrophy, myasthenia gravis or myotonia)	

### Informed consent

Written informed consent for study participation will be obtained 1–7 days after screening for eligibility. Participants will be informed that successful completion of the baseline tests is required before randomisation to an intervention group.

### Eligibility criteria

The diagnosis of stroke will be confirmed by a neurologist before admission to our neurological rehabilitation hospital. A previous stroke is no reason for exclusion. Participants will be in the subacute phase (within three months after stroke) and will have a Barthel Index (BI) of at least 30 points. If the BI is 85–100 points (mild stroke), the patient will be included at least 24 h after stroke. If the BI is < 85 points (moderate stroke) the patient will be included at least seven days after stroke. They will be able to sit free for 30 s (sitting balance score of at least 2 points) and they will be able to lift the affected hand from their lap to a desk standing in front of them. They will have a Medical Research Council Score of 2–4 for shoulder abduction, elbow and finger flexion. The passive range of motion (ROM) will allow a touch of finger tips and thumb as well as submaximal finger extension. Patients will achieve an Apraxia Screen of TULIA Score of ≥ 5 (for details, see Table 1).

### Outcome measures

The primary efficacy endpoint is grip strength. We will measure the handgrip strength for the affected hand (in kg) using a hand dynamometer (Camry Digital Hand Dynamometer; Camry Scale Store, City Industry, CA, USA). Sunderland et al. found a high correlation between grip strength and arm function [28]. Measuring grip strength is simple, low time-consuming and predictive for recovery of the arm. Therefore, we set grip strength of the affected hand as our primary outcome assessment.

The secondary endpoints include tests which assess impairments, activities and participation for patients with stroke. We will measure functional changes in muscle tone and reflex activity of the Golgi Apparatus using the Modified Ashworth Scale (MAS) with a score in the range of 0–5 score [29]. We will measure muscle strength of the upper limb (shoulder, elbow and wrist) using the Motricity Index (MI) with a score in the range of 0–100 [30]. We will use the Fugl-Meyer Assessment for the upper limb (FMA-UL) with a score in the range of 0–66 [31, 32] to measure sensorimotor impairments and recovery. We will measure the dexterity of the affected arm using the Box and Block Test (BBT) with 0–150 cubes/min [32]. The Goal Attainment Scale (GAS) with a score in the range of − 2 to + 2 is a measurement which allows the formulation and standardised evaluation of individual goals with the patient [33]. We will use this Scale to measure participation.

These measurements are frequently used in research and/or clinical practice dealing with this patient group. Figure 2 gives a detailed overview of the variables we will use and the data collection schedule.

### Assessment of safety and adverse events

During the training, the examiner monitored heart rate (HR) and rating of perceived exertion (RPE) according to Borg’s 20-point scale [34, 35]. Different intervention types in stroke rehabilitation have an impact on the cardiorespiratory system [36]. HR monitoring can be used to measure cardiorespiratory responses during training [36, 37]. The maximal HR during training should not be higher than the age-predicted maximal HR of 220 minus age to avoid physical stress [38]. We will monitor HR to assess training effects on the cardiorespiratory system and to avoid cardiovascular stress caused by training. In addition to the HR, the subjective exertion sensation should be assessed with the Borg Perceived Exertion Scale to prevent severe physical stress [39, 40]. The rating of perceived exertion allows conclusions about training intensity [41]. For example, a perceived exertion of 16 points represents a training intensity of 80% of the one repetition maximum and 10 points represents an intensity of 40% of the one repetition maximum. Thus, this information can be used to monitor training intensity and to check if the intensity exceeds the expected 16 points Borg’s rating of perceived exertion scale.

There is a risk for heavy physical strain or shoulder pain of the hemiparetic arm due to a high weight load in strength training. In order to ensure the safety of the study participants, the following risk-management processes will be performed:

1. We will follow clearly defined eligibility criteria to include only patients who do not have severe heart diseases or have pain in the affected arm.
2. We will periodically monitor HR and rating of perceived exertion during the training.
3. The training will be guided by qualified staff (physiotherapists).
4. AEs such as self-reported fatigue, a decline in performance of 30%, pain, dizziness, amentia or impaired consciousness, sickness, dyspnoea, tinnitus, angina pectoris, muscle soreness, swelling and decrease in compliance to training will be continuously recorded.

After each intervention, the treating physiotherapist will record the presence of AEs. During the intervention phase and at the final assessment the following parameters will be systematically recorded: cardiovascular or cerebrovascular events; referral to an acute hospital; and death.

### Standardisation of assessments

Blinded assessors have systematically undergone competency training to ensure standardisation of data collection methods. This included theoretical learning about the assessments, demonstration and practice on volunteers (patients with stroke in our rehabilitation hospital) under the supervision of the study’s clinical research coordinators. The success of blinding will be formally tested by asking the outcome assessors to guess the study group assignment of patients after the end of the study period and comparing these responses to what would we expect by chance.

### Data collection

Pseudonymised outcome data are collected by blinded assessors at baseline (t0) and after three weeks of therapy (t1) (see Fig. 2). Pseudonymised outcome data will be entered in a spreadsheet by a masked researcher who has no knowledge of identifiable participant information or treatment assignment.

### Randomisation and concealment of allocation

Each study participant will be randomly assigned to one of two intervention groups, either the low-intensity training group or the high-intensity training group. Allocation will be concealed within an opaque sealed envelope. The computer-generated random allocation sequence list will be carried out using a random-number generator (randomizer.org). Assignments will be enclosed in sequentially numbered, opaque, sealed envelopes and stored at the central study centre. The persons who assess eligibility, obtain informed consent and enrol patients in the trial (SH, CH) have no knowledge of group assignment (Fig. 2). After recruitment and after the initial assessment, the appropriate numbered, opaque, sealed envelope will be opened and the randomisation information will be given to the patient and therapists (but not to the outcome assessors). We will not include further potential covariates in our stratification. However, we will test the influence of different covariates statistically on the main effect of the primary outcome.

### Interventions

Patients will be randomly assigned to either the low-intensity training group or the high-intensity training group. Tables 2 and 3 show the training programmes. Patients of both groups will receive, in addition to standard treatment, functional arm training three afternoons per week for three weeks. Each training session will last for 60 min, including a 5-min warm-up. The warm-up will prepare the muscles with ball games. This additional training programme contains five standardised and systematically augmented exercises arranged in a circle. The training includes unilateral, active and functional exercises, which will be practised from a sitting position:

1. lifting objects from the lap to a high desk;
2. pulling a resistance band from vertex high to the lap;
3. pulling a mineral water crate on a desk from the unaffected to the affected side;
4. lifting objects over a wooden block with the elbow placed on the table (like an arm-wrestling position);
5. and pulling a laundry bag which is laying on an exercise mat with a rowing motion.

**Table 2:** Overview of the schedule for increasing intensity in the high-intensity arm training group

Session	Exercises	Weight	Repetitions	Increase of weight (if participant establishes 15 repetitions)	Sets	Rest between sets (s)
1	5	80% of 1RM	10–15	By 1 unit of weight	3	120
2	5	As in previous session + increase	10–15	By 1 unit of weight	3	120
3	5	As in previous session + increase	10–15	By 1 unit of weight	3	120
4	5	As in previous session + increase	10–15	By 1 unit of weight	3	120
5	5	As in previous session + increase	10–15	By 1 unit of weight	3	120
6	5	As in previous session + increase	10–15	By 1 unit of weight	3	120
7	5	As in previous session + increase	10–15	By 1 unit of weight	3	120
8	5	As in previous session + increase	10–15	By 1 unit of weight	3	120
9	5	As in previous session + increase	10–15	By 1 unit of weight	3	120

*1RM* one repetition maximum

**Table 3:** Overview of the schedule for increasing intensity in the low-intensity arm training group

Session	Exercises	Weight	Repetitions	Increase of repetitions	Sets	Rest between sets (s)
1	5	40% of 1RM	10	n. a.	3	120
2	5	40% of 1RM	11	By 1	3	120
3	5	40% of 1RM	12	By 1	3	120
4	5	40% of 1RM	13	By 1	3	120
5	5	40% of 1RM	14	By 1	3	120
6	5	40% of 1RM	15	By 1	3	120
7	5	40% of 1RM	16	By 1	3	120
8	5	40% of 1RM	17	By 1	3	120
9	5	40% of 1RM	18	By 1	3	120

*1RM* one repetition maximum

The resting time will be 120 s after each exercise and set. During this time, the participant changes position for the next exercise. Therefore, two or three people can train at the same time. The resting time between training sessions should be 24 h. Shorter resting times between training sessions are possible in exceptional cases, e.g. if a training is cancelled. The training will be led by qualified and instructed therapists and intensity will be increased progressively according to Tables 2 and 3. The therapists will have to guide and motivate the participants without an active intervention in the exercise (hands-off), except when danger is imminent.

In the first training, participants will be requested to do the exercise without any weight to find out the maximum ROM. They will attempt to reach this ROM in every repetition or to increase it. Each exercise’s one repetition maximum (1RM) will be tried out for this maximum ROM in as few as possible trials.

### High-intensity training group

The workload in the arm strength training group will be 80% of 1RM, examined at the beginning of the first training session. Participants will do three sets of ten repetitions of each exercise. Resistance will be increased gradually once the participants are able to perform 15 repetitions according to Table 2.

### Low-intensity training group

The workload in the low-intensity training group will be 40% of 1RM, examined at the beginning of the first training session. Participants will also do three sets of an increasing number of repetitions of each exercise. In the first training session, they will perform ten repetitions, one more in each following training session, with up to 18 repetitions in the final training session according to Table 3. The resistance will not be changed.

We chose a low-intensity progressive training to compare two true active and potentially effective interventions. One intervention increases by weight and the other by repetitions. This low intensity was often used in therapeutic studies for stroke patients [14, 42].

If the grasp of the objects is not possible, a wrist cuff can be used to fix the grip. The therapists will document ROM, repetitions and intensity for each exercise in each session. If a training is cancelled (because of illness or other liabilities), it will be repeated as soon as possible.

The therapy session will be adjusted, interrupted or terminated if the patient (using continuously monitoring):

1. has pain with a numeric rating scale score > 5;
2. has a body temperature > 37 °C,
3. has > 16 points of Borg’s rating of perceived exertion scale;
4. or has a decline in performance of 30%.

The adherence of the training programme will be documented and reported. All single exercises in the arm training programme are well-known and used routinely under different circumstances for the target patients in our clinic. Our additional training programme with systematically augmented exercises is, however, not described in the medical literature yet. For this reason, we underwent a pilot training programme testing the feasibility of the arm training programme in our clinic with all above-mentioned criteria. In five patients, we found that this pilot training programme might be feasible without any AEs.

Both groups will be supervised by one of two experienced physiotherapists not involved in assessment. To control for co-interventions (standard therapy for the upper limb) during the intervention phase of the study (t0 to t1), we will extract information of the total duration and content of both standard and additional therapy from the daily and weekly documentation of our clinic. Content will be described for each 10-min block.

### Criteria for discontinuing or modifying allocated intervention in both groups

Therapy sessions will not occur if any of the following conditions are present:

1. body temperature > 37 °C;
2. repeated pain with a numeric rating scale score > 5 (no pain relief during adjusted training);
3. > 16 points of Borg’s rating of perceived exertion scale in two training sessions;
4. a decline in performance of 30% in two following therapy sessions;
5. rehabilitation team perception that therapy is not appropriate despite absence of the above criteria;
6. refusal of consent;
7. decreased alertness and compliance cannot be handled by the therapist;
8. or if training is not possible for one week, without the chance to catch up.

### Additional information

The standard treatment for the affected arm includes mobilisation, stretching, therapeutic positioning, arm and hand motor training, strengthening exercises, mechanical assisted training, functional training and task-oriented training. The standard treatment will be approximately 30 min of individual physical therapy three times a week and 30 min of occupational therapy three times a week. The duration of therapy will be kept equal in both intervention groups. The total time spent in rehabilitative therapy will be recorded by so-called usual care intervention logs to report the number of physiotherapy and occupational therapy sessions received in the three weeks of the study (as part of the usual care treatment).

For the description of the population and for interpretation of the results, the following data will be documented: age; sex; pedestrian or wheelchair user; time since onset; type of stroke; affected side; number of strokes; Mini-Mental State Examination (MMSE); BI; neglect; pusher syndrome; impaired depth sensitivity; aphasia; apraxia; shoulder-hand syndrome; depression; and information about medication.

### Sample size

Based on the study by da Silva et al., we assume a mean difference of 10 kg in the intervention group and a mean difference of 8 kg in the control group with a standard deviation (SD) of 3 kg [11]. We assume an alpha level of 5% and a statistical power of 80% (beta = 20%) using a two-sample t test for mean differences. We used the G*Power Version 3.1.9.2 power and sample size programme of the university of Kiel, Germany for our sample size calculation (‘G*Power’, 1992–2014). According to the programme, we will need 37 patients per study arm. Because we expect a 5% dropout rate, a total of 78 patients (39 per study arm) would be enrolled to ensure 37 individuals in both intervention arms.

Sample size calculations can be based on clinical meaningful differences, on between-group comparisons of similar studies or conveniently on both.

The study by Lang et al. is the only one which examined the minimal clinical important difference of grip strength; its results are limited by a small sample size and wide standard deviations [33]. There are no further studies which verified these results.

Because of these limitations we decided to use the results of another study which assessed between-group differences in grip strength after upper limb strength training [6].

### Recruitment

Based on our sample size calculation, 78 patients will be recruited from our rehabilitation hospital over a period of nine months. We will screen all patients consecutively from our rehabilitation hospital of the Asklepioskliniken Schildautal in Seesen, Germany and recruited patients who meet our eligibility criteria for our RCT. The research staff will screen lists of patients every second day to recruit continuously and will monitor recruitment. Eligible patients will be screened and receive oral and written information about the study from a researcher later. After signing the informed consent, demographic and clinical characteristics will be measured (baseline assessment, t0). Patients will then be measured three weeks after baseline (t1). Recruitment statistics for every week will be discussed to improve the recruitment procedure. Based on this, the research team discusses updates to recruitment and retention strategies, if necessary. Based on the recruitment rate of a previous study, we find it reasonable to recruit the anticipated sample size for our trial within nine months [18].

### Statistical methods

We will conduct both intention-to-treat and per-protocol analyses, with the intention-to-treat analysis being always the primary analysis for the primary and secondary outcomes.

Our descriptive statistics will include means and SD for continuous variables, and the number and proportions for categorical variables as appropriate [43]. We will compare the two intervention groups at baseline regarding characteristics and demographics, using two-tailed Student’s t tests or Fisher’s exact tests, as appropriate. The alpha level will be set to 0.05 for all comparisons. To avoid multiplicity, we will use a Bonferroni alpha adjustment for multiple comparisons of the same outcome. Skewed data will be analysed with non-parametric test alternatives (e.g. we will use the Mann–Whitney U test instead of Student’s t tests if data are skewed). For the primary endpoint, the grip strength, we will test the hypothesis that there is a significant difference after three weeks of intervention between groups. We will consider a minimum difference of 5 kg of the grip strength between groups as being clinically important [44]. We will use IBM SPSS Statistics Version 23.0.0.0 for all statistical procedures (IBM Corporation, Armonk, NY, USA). No interim analysis will be conducted.

### Adverse event monitoring and reporting

All AEs will be carefully monitored at every level of the study. All AEs are reported immediately to the responsible physician and the research staff is informed of all AEs. The research team will be responsible for data safety and for the confidentiality of recruited patients. The confidentiality of participants will be granted by using pseudonymised ID lists for all recruited patients. All patient data will be maintained and stored confidentially on a separate server.

The trial will be stopped:

- if there is an important difference in safety between the groups in one direction,
- if one of the interventions is associated with unexpected excessive adverse effects such as uncontrollable pain or immoderate high points of Borg’s rating of perceived exertion scale, or there are excessive withdrawals;
- if the study recruitment is unsuccessful (e.g. three months without any recruitment);
- or if other situations occur that might justify stopping this trial.

### Ethics and dissemination

This study will be conducted in accordance with the Helsinki Declaration. The study is non-invasive, imposes no additional risk on patients, seems to have enough power to detect meaningful determinants and our protocol has been approved by the medical ethical committee (ethical vote ‘Universitätsmedizin Göttingen’ Germany; 23/3/17). Furthermore, written informed consent is obtained from all participants or, if necessary, from a legal guardian. We registered our study on 26 May 2017 before enrolling the first patient in the trial at the German Clinical Trials Register (https://www.drks.de/drks_web/navigate.do?navigationId=trial.HTML&TRIAL_ID=DRKS00012484) with the identifier DRKS00012484. We plan to disseminate the results of this study to scientists, health professionals and the general public by publication in national and international peer-reviewed journals, as well as by presentations at conferences and meetings with clinicians working with patients with stroke. Authorship will follow the recommendations of the International Committee of Journal Editors for authorship. No professional writers will be employed.

## Discussion

The results of this trial might help to close a research gap for high-intensity arm strength training of people with subacute stroke. Only two RCTs investigated progressive resistance arm training in subacute stroke patients [45]. Both focused on changes in arm function [17, 18] but did not find advantages for strength training compared to standard treatment, additional physiotherapy or functional training. However, the validity is limited because of methodological deficits – there were no blinded assessors [17] or study groups where inhomogeneous [18]. If strength training was used for patients with stroke, they were often trained with low or moderate intensities [11, 14, 42] or the intensity and progression where imprecisely described [12, 13, 15, 17, 46]. This will be an adequate powered and funded study to determine whether an intensive strength training for people with stroke is beneficial in the subacute care of these patients.

### Limitations

A limitation of this study is the lack of follow-up assessment. Long-term changes following the progressive arm strength training cannot be evaluated. Including patients with mild aphasia, apraxia or neglect comes close to realistic neurological rehabilitation but could also decline therapeutic effects. One could argue that the lack of a true control group, e.g. of standard or no additional physiotherapy, might be a limitation of our trial protocol. There is a need for effective therapy to maximise and accelerate the recovery of patients with stroke. Therefore, we decided to compare two potentially capable and reasonable active physical rehabilitation interventions. Another limitation is that this trial is planned to be carried out as a single-centre study. Future RCTs should try to recruit several centres and hospitals to get a representative sample.

## Trial status

Patient recruitment began on 29 May 2017 and is expected to continue for two and a half years.

## Additional file

Additional file 1: SPIRIT 2013 Checklist: Recommended items to address in a clinical trial protocol and related documents. (DOC 124 kb)

## Abbreviations

1RM: One repetition maximum
ADL: Activities of daily life
BBT: Box and Block Test
BI: Barthel Index
FMA-UL: Fugl-Meyer Assessment for the upper limb
GAS: Goal Attainment Scale
HR: Heart rate
MAS: Modified Ashworth Scale
MI: Motricity Index
MMSE: Mini-Mental State Examination
MRC: Medical Research Council
RCT: Randomised controlled trials
ROM: Range of motion
RPE: Rating of perceived exertion
SD: Standard deviation
t: Time point
